# Production and Characterization of Novel Photocatalytic Materials Derived from the Sustainable Management of Agro-Food By-Products

**DOI:** 10.3390/molecules31020300

**Published:** 2026-01-14

**Authors:** Christina Megetho Gkaliouri, Eleftheria Tsampika Laoudikou, Zacharias Ioannou, Sofia Papadopoulou, Vasiliki Anastasia Giota, Dimitris Sarris

**Affiliations:** Laboratory of Physico-Chemical and Biotechnological Valorization of Food By-Products, Department of Food Science and Nutrition, School of the Environment, University of the Aegean, Mitrop. Ioakeim 2, 81400 Myrina, Greecesofiapdl244@outlook.com (S.P.); dsarris@aegean.gr (D.S.)

**Keywords:** juice industry by-products, photodegradation, carbon-TiO_2_, photocatalyst

## Abstract

Porous photocatalysts from agricultural waste, i.e., apricot and peach shell, with titanium dioxide were prepared by a carbonaceous method, the adsorption and photocatalytic degradation and its kinetics about methylene blue (MB) were studied systematically. The properties of the prepared composite sorbents were characterized using Brunauer–Emmett–Teller, surface area, scanning electron microscopy, and energy dispersive spectroscopy analyses. Several key factors, including radiation, pH, temperature, initial MB concentration, contact time, and sorbent dosage, as well as photocatalytic activity were investigated. All the waste-TiO_2_ adsorbents showed improved adsorption and photodegradation performance compared to commercial charchoal-TiO_2_. The produced materials presented high specific surface areas especially those derived from apricot shell-TiO_2_ with a combination of type I and IV adsorption isotherms with a hysteresis loop indicating micro and mesopore structures. In addition, under UV radiation, the composite sorbents exhibited greater MB removal efficiency than non-radiated composite sorbents. The examined conditions have shown the best MB adsorption results at pH greater than 7.5, temperature 30 °C, contact time 120 min, initial concentration 0.5 mg/L MB, and sorbent dosage equal to 2.0 g/L C/MB. The total removal rate of MB is 98.5%, while the respective amount of commercial charcoal-TiO_2_ is equal to 75.0%. The kinetic model that best describes the experimental data of MB degradation from the photocatalytic materials is the pseudo-second order model. In summary, this work highlights the effectiveness and feasibility of transforming agricultural waste into carbonaceous composite sorbent for the removal of cationic dyes from wastewater. Future work will involve scaling up the synthesis of the catalyst and evaluating its performance using bed reactors for industrial processes.

## 1. Introduction

Fruits and vegetables are among the most consumed products worldwide, accounting for more than 42% of total food waste [1,2]. Pulp, peel, seeds, skin, pomace, husks, pods, and stems are some of the by-products produced in the fruit and vegetable industry, representing the majority of agro-food by-products. These by-products are available in large quantities and can be easily recovered and reused [3]. In Greece, the apricot industry is located in Peloponnese (South Greece) and in Halkidiki, while the peach production is in Imathia, both latter regions in North Greece. The apricot production varies between 60.000 and 80.000 tons/yr according to the weather conditions, while the production of peaches is approximately equal to 380.000 tn/yr [4,5]. The European Union (EU) has adopted an action plan to eliminate food waste, using the circular economy as a method. It includes the reduction, reuse, recovery, and recycling of materials and energy to increase the value of goods, materials, and resources. Therefore, the utilization of by-products is an important factor in achieving the circular economy, with the aim of both reducing and effectively managing waste [6].

The rapid increase in the world population combined with the constant progress of industrial processes has led to an increase in the discharge of chemical pollutants resulting in the degradation of water quality [7]. The most widespread organic dye discovered in wastewater is methylene blue (MB), mainly due to its widespread use in several industries, such as food, paper, plastic, leather, and textile [8]. Methylene blue (MB) is a common cationic phenothiazine dye. It serves as a redox indicator, as well as a biological dye, due to its strong adhesion and good stability [9]. As has been shown, even at very low concentrations, the presence of MB in water bodies can have negative effects on human health, causing various diseases. Therefore, addressing the pollution caused by this pigment is of paramount importance [10].

Activated carbon (AC) is a porous, amorphous form of carbon known for its strong adsorption capacity, making it highly effective for use in wastewater treatment [11]. The continuous contamination of water by emerging pollutants and the need for more efficient and sustainable treatment methods have prompted the exploration of advanced materials and technologies, such as adsorption and photocatalytic degradation [12]. Adsorption is the phenomenon in which molecules from one phase (liquid or gas) are concentrated and retained on the surface of a solid. That is, it is a process that takes place at the interface between two different phases, where a thin layer of molecules is formed. There are two types of adsorption: physical adsorption, which is a connection through Van der Waals forces between substrate and adsorbent material, and chemical adsorption, which is the formation of a chemical bond between substrate and adsorbent material [13].

Although adsorption is used to decolorize MB dye, the mineralization of organic pollutants is not always achieved [14]. Degradation of MB is required for complete mineralization after adsorption on the photocatalyst, as photocatalysis has the unique advantage of converting toxic components into non-toxic ones, such as water (H_2_O), carbon dioxide (CO_2_), and nitrogen (N_2_), among others [15]. Photocatalysis uses sunlight and water as its main inputs, making it a renewable option [16]. However, the difficult reuse of photocatalysts and the low absorption rate of solar energy are some disadvantages that recent studies have ameliorated [7]. Photocatalytic degradation is generally preferred due to its efficiency in processing, wide application, absence of secondary pollution [17], but also the utilization of light radiation for the breakdown of pollutants [18].

Carbon-based metal oxide nanocomposites are a promising solution for the treatment of contaminated water [7]. Among the various semiconductor photocatalysts, TiO_2_ exhibits excellent chemical stability, cost-effectiveness, non-toxicity, and mild conditions of use. Also, when exposed to ultraviolet light, it produces particle pairs (electrons/holes) that cause chemical reactions, breaking down organic pollutants and removing harmful substances from the environment [19]. However, TiO_2_ particles are finely aggregated during dye treatment processes in wastewater, which makes it difficult to recycle and reuse the material. Consequently, its reuse rate is low, while its practical application cost is high. Furthermore, the easy binding of photogenerated electron–hole pairs in TiO_2_ prevents the outward diffusion of charge carriers, thus slowing down the degradation reaction between semiconductors and organic matter [20].

In this research, apricot and peach kernels with the addition of TiO_2_ were utilized to produce novel photocatalytic carbon materials. The aim was the characterization of these materials through scanning electron microscopy with energy dispersive spectroscopy (SEM-EDS) and BET (Brunauer–Emmett–Teller) porosimetry, their applications as water purification filters and the sustainable management of the regions of Northern and Southern Greece by reducing the excess amount of juice industry by-products.

## 2. Results and Discussion

### 2.1. Characterization of the Produced Materials

The moisture content of the by-products, i.e., apricot and peach shells, after 2 h in the air-dry oven at 103 ± 2 °C are equal to 9.86 ± 0.20 for apricot shell and 14.13 ± 0.40% for peach shell. It seems that peach shells present higher amounts of moisture compared to apricot by-products. Moreover, the burn-off (%) amount of the pyrolyzed materials is equal to 73.02 ± 0.52% for apricot shells and 79.67 ± 0.85% for peach stone. After the impregnation of the four photocatalytic materials, i.e., commercial charcoal, carbon derived from apricot shell, and carbon derived from peach shell, and mixtures of them (50/50% *w*/*w* apricot/peach shell) with TiO_2_ solution, the percentage of weight loss after the oven was 76.40 ± 0.95% for CCTi, 77.74 ± 0.65% for ACTi, 73.09 ± 1.65% for PCTi, and 75.11 ± 0.83% for APCTi, respectively.

**Figure 1:**
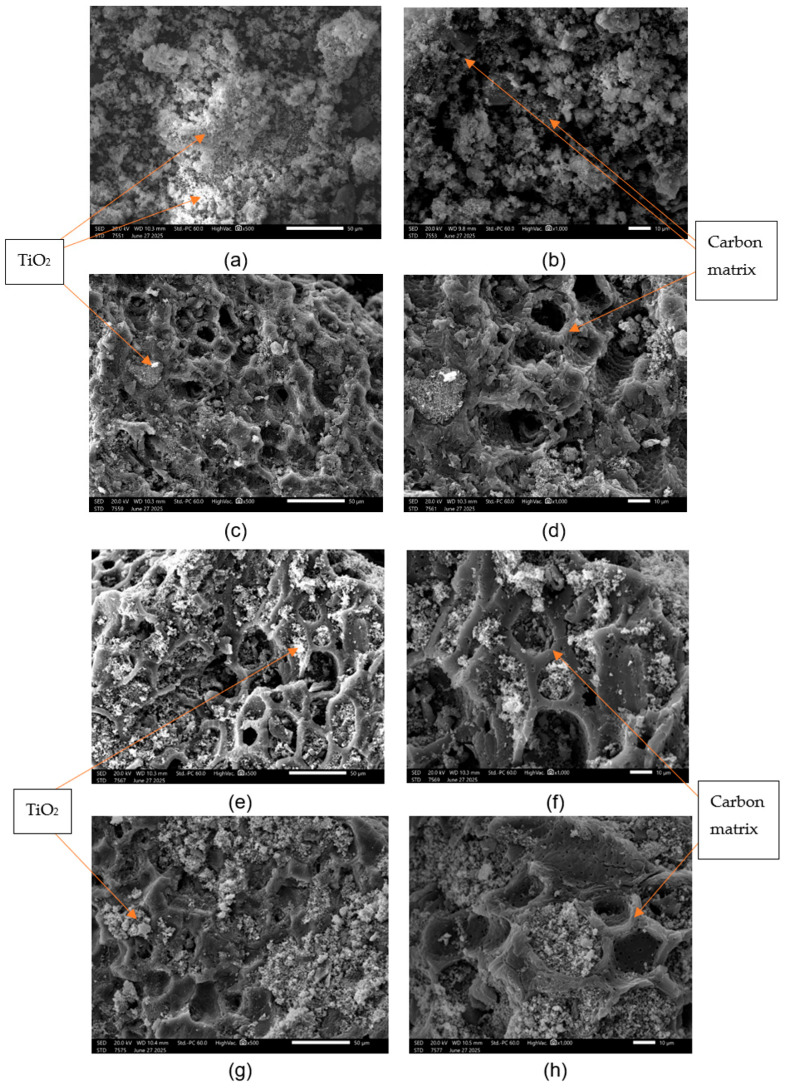
SEM images of the photocatalytic materials after the addition of TiO_2_: (**a**,**b**) commercial charcoal (CCTi), (**c**,**d**) carbon derived from apricot shell (ACTi), (**e**,**f**) pyrolyzed carbon derived from peach shell (PCTi), and (**g**,**h**) pyrolyzed carbon derived from apricot and peach shell in a proportion of 50/50 *w*/*w* (APCTi) at two different magnifications 500× and 1000×.

Scanning electron microscopy of the raw pyrolyzed materials derived from agricultural by-products is shown in Figure S1 (Supporting Information). The images are from 500× and 1000× resolution where SED refers to a secondary electron detector, which captures secondary electrons to create images showing the surface topography. From the SEM images it can be seen that the pyrolyzed carbon derived from apricot (Figure S1a,b), peach shells (Figure S1c,d), and mixtures of them (Figure S1e,f) presents larger particles, in some cases exceeding 50 μm. The surface morphology of carbon materials has even cavities (tetrahedral or pentahedral structures) and ridges which means that they exhibit a porous structure. The samples are structured material with the inclusion of macropores with a diameter of 10–20 μm. The scanning electron images of carbon-doped TiO_2_ materials are presented to Figure 1. Comparing the commercial charcoal-TiO_2_ (Figure 1a,b) with agro-food by-product carbon-TiO_2_ (Figure 1c–h), it is evident that commercial charcoal includes more compact structures surrounded by white regions, which belong to TiO_2_. The charcoals derived from apricot (Figure 1c,d), peach shells (Figure 1e,f), and mixtures of them (Figure 1g,h) present different particle sizes compared to commercial charcoals, and well-formed carbon structures (tetrahedral or pentahedral structures) fulfilled with TiO_2_ (white regions). SEM images of commercial activated carbon derived from coconut shells show the presence of many thin layers with rudimentary pores among them [21]. The materials present many small cavities across the surface showing a well-formed pore structure [21].

Based on the EDS analysis (Table S1) it appears that the dominant element is carbon when no TiO_2_ impregnation is performed. The elemental carbon of the pyrolyzed materials (AC: apricot shell, PC: peach shell, APC: apricot-peach shell mixture) presents high values (>88%) indicating that the carbonization of the materials is successful. The C/O ratios rank the pyrolyzed materials in the following order: APC > PC > AC. In the case of the pyrolyzed carbons with Ti (Table 1), it appears that the TiO_2_ impregnation was successful given that the elemental analysis shows that the Ti/C ratios range from 20.3 (APCTi) to 4.0 (PCTi). All materials embedded with TiO_2_ solution exhibit both TiO_2_-coated areas mentioned with the subscript a in Table 1, representing the white regions of Figure 1, and smaller uncoated pyrolytic carbon areas with the subscript b in Table 1, representing the gray regions of Figure 1. In the produced by-product materials, TiO_2_ molecules cover the cavities of the pyrolyzed coals as seen in the SEM images (e.g., Figure 1c,e,g). According to Table 2, commercial pyrolyzed coals (CCTi) present more impurities than coals derived from by-products. Commercial coals present high amounts of elemental Fe, Ca, K, Si, and Al and smaller amounts of S, Mg, Cl, and Na. On the contrary, the impurities in the carbons derived from by-products mainly present elemental K and smaller amounts of Ca, Si, and P, which usually come from the processing of the raw material. The presence of K in the samples of pyrolyzed coal of apricot and peach kernels is a result of the use of fertilizers and spraying with chemical preparations from the trees.

Impurities in the carbon matrix influence the photocatalytic activity of carbon/TiO_2_ composites by creating defects, narrowing band gaps (for visible light use), improving adsorption, and providing active sites, resulting in the amelioration of photocatalytic activity. On the contrary, they can also limit the photocatalytic performance by blocking sites or causing unwanted side reactions, depending on the specific impurity type and reaction conditions. More specifically, the presence of Fe, Mn, and Ca impurities during the hydrothermal carbonization of carbon derived from crop straw (HTCS) catalyzes the formation of HTCS and promotes the photocatalytic removal of sulfamethoxazole (SMX) on HTCS [22]. Comparing the photocatalytic behavior of three HTCS materials with different quantities of trace elements, it seems that the increase in the impurity content of the composite leads to an increase in SMX removal. The presence of Mn and Ca impurities change the hydrothermal carbonization carbon structure, presenting more C=O functional groups and furan rings polymerization leading to electron deficient connection sites for SMX to form HTCS-SMX complex, and electrons were transferred from the HOMO state of SMX to the CB state of HTCS under visible light illumination. Another study [23] has examined the photocatalytic degradation of Rhodamine B (RhB) from Fe^3+^-doped alkalized carbon nitride. It seems that g-C_3_N_4_ material doped with Fe^3+^ shows better photocatalytic activity in the degradation of RhB compared to pure carbon nitride due to the bandgap width decrease and the low required energy for the transition of photogenerated electrons from the valence band (VB) to the conduction band (CB). A similar study [24] has shown the use of salicylic acid-pretreated artificial converter slag rich in Fe and Ca ions for the successful preparation of Fe(III)-doped HAp (Fe(III)-HAp) using a modified hydrothermal reaction. According to the results, the Fe(III) doping with the formation of oxygen vacancies narrowed the bandgap of HAp and broadened the light response range, ameliorating its photocatalytic activity, i.e., better xanthate photooxidation under visible illumination. Al impurities contained in artificial converter slag may affect the morphology and location of active metal ions (like Fe) within composite structures, influencing overall catalytic behavior. Different amounts of K ions were incorporated into TiO_2_ examining its photocatalytic activity towards acetaldehyde. It seems that small quantities of K ions, i.e., 0.008% w. on TiO_2_ ameliorate photocatalytic activity of acetaldehyde under dry conditions while higher K concentrations led to lower photocatalytic activity due to the formation of separate and less active K-compound structures on the TiO_2_ surface [25]. According to Table 2, comparing the produced composites, it seems that the existence of Ca ions in ACTi and the low amount of K ions enhances the photocatalytic activity of the composite compared to APCTi and PCTi. The lower K concentration of APCTi compared to PCTi ameliorates the photocatalytic activity of APCTi, as is referred to above. Finally, CCTi presented the highest amount of K ions compared to all the other composites, indicating the lowest photocatalytic activity despite the small amount of Fe and Ca ions.

The basic characteristics of the pore structure of all adsorbents are given in Table 3. Comparing the specific surface area (S_bet_) of the materials, it seems that the increase in the S_bet_ follows the order: CCTi < PCTi < APCTi < ACTi. According to Table 3 and Table S2, the addition of TiO_2_ led to significantly higher specific surface area of embedded carbons, i.e., ACTi, PCTi, and APCTi, compared to the carbonaceous materials (AC, APC, PC). in accordance with the classification adopted by the International Union of Pure and Applied Chemistry (IUPAC) [26], the adsorbent pores are classified into three groups: micropore (diameter < 2 nm), mesopore (2–50 nm), and macropore (>50 nm). The adsorption average pore width according to BET method shows that the carbonaceous materials derived from agro-food by-products mainly exhibit pores of 2.25–2.38 nm in diameter and are classified on the border between micro- and mesopores, while the pore sizes of carbonaceous materials without TiO_2_ range from 7.5 to 15.3 nm (Table S2). According to BJH method, the average pores of the materials are classified as mesopores. Moreover, comparing the total pore volume (TPP) and the t-plot microporous volume (TMV) of the materials, it seems that the higher TPP are presented to ACTi, where TMV are equal to 51.0% of TPP, while CCTi appears as the lower TPP, where TMV are equal to 9.0% of TPP. All the other materials present intermediate values. Comparing Table 2 and Table 3, it seems that the highest specific surface area is presented to adsorbents with the lowest content of carbon and the highest content of oxygen, e.g., ACTi has the highest S_BET_ and oxygen and lowest carbon compared to APCTi and PCTi. Similar results are mentioned elsewhere [27], where the textile sewage sludge activated carbon adsorbent (TSSAC) with the highest BET surface area has shown the lowest carbon and sulfur content and the highest oxygen content. The increase of oxygen content in photocatalytic carbon ameliorates the specific surface area and improves the photocatalytic activity due to the introduction of additional -O- groups such as carbonyl groups (-CO) and -OH functional groups. These hydrophilic groups are responsible for the material’s adsorption capacity producing a more porous morphology in carbons. The higher specific surface area leads to more available sites on the catalyst’s surface where the photocatalytic reaction can occur. The oxygen doping improves also the charge separation efficiency of the carbon, a crucial parameter for reactive oxygen species generation and photocatalysis.

**Figure 2:**
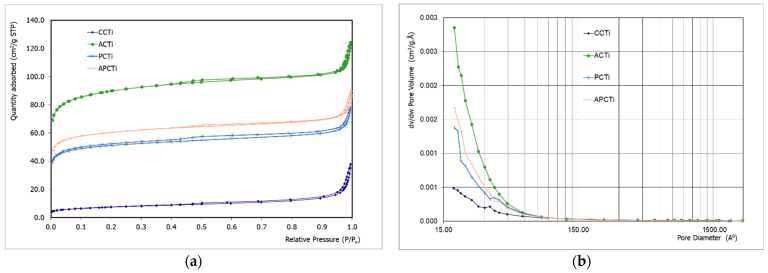
(**a**) The N_2_ adsorption–desorption isotherm and (**b**) the pore size distribution (PSD) based on the BJH method of the prepared samples.

The porous features of the adsorbents are analyzed using nitrogen isothermal adsorption/desorption measurements and pore size distributions (Figure 2). All samples show a combination of type I and IV adsorption isotherm with a hysteresis loop indicating micro- and mesopore structures [10,28]. The hysteresis loop reflects the connectivity of various sized pores, and the adsorbent develops considerable micropores and mesopores. Hysteresis can be produced by the presence of open pores, such as cylindrical pores, wedge-shaped pores, and parallel plate pores, especially thin bottleneck pores, but not closed pores. It is effectively caused by capillary condensation and evaporation at the region for P/P_0_ > 0.45 in large pores with diameters greater than 40 Å and by the tensile strength effect for small pores with diameters less than 40 Å [29]. The tensile strength effect leads to a decrease in the desorption branch, particularly around P/P_0_ = 0.45, causing the closure of the hysteresis loop. The hysteresis loop is mainly correlated with four pore types according to Labani et al. [30].

Type A hysteresis is attributed to cylindrical pores; type B is associated with slit shaped pores; type C and D hysteresis is produced by wedge-shaped pores and type E hysteresis has been attributed to bottle neck pores [30]. Consequently, based on the classification by Sing [10], the pores in the experiment can be interpreted as the narrow slit-type pores. The pore size distribution measurements (Figure 2b) also show that the production of photocatalytic materials based on apricot and peach shell embedded to TiO_2_ solution show that the highest pore volume of the materials is presented by pores on the border between micro- and mesopores. Commercial charcoal embedded with TiO_2_ solution shows that the pore volume is higher than pore diameter in the region of mesopores (2 < d< 50 nm).

Other studies have shown the production of the photocatalytic multilayered NiFe LDH/activated carbon that was characterized by BET analysis showing surface area equal to 141.2 m^2^/g, average pore size equal to 20.7 nm, and total pore volume equal to 0.62 cm^3^/g [10]. The field emission scanning electron microscopy (FE-SEM) and the high-resolution transmission electron microscopy (HR-TEM) have indicated a nanoflower sphere-like shape with radially organized NiFe LDH nanosheets. EDS analysis shows the presence of carbon (34.54% *w*/*w*), oxygen (36.15% *w*/*w*), Al (0.19% *w*/*w*), Si (0.11% *w*/*w*), Fe (6.89% *w*/*w*), and Ni (22.12% *w*/*w*). Activated carbon-based nanocomposites (NCs) loaded with zirconium dioxide and cerium dioxide nanoparticles (ZrO_2_/CeO_2_ NPs) were successfully synthesized for the effective elimination of methylene blue (MB) and tetracycline hydrochloride (TCH) [31]. The materials were characterized by BET analysis for surface area, pore volume, and pore size analysis showing that the BET surface area was equal to 501.75 m^2^/g, the average pore size was 1.89 nm, and the total pore volume was 0.47 cm^3^/g measured by a N_2_ adsorption–desorption isotherm at 77.35 K and P/P_0_ = 0.99. The AC-ZrO_2_/CeO_2_ NCs exhibit a mixed type I and type IV characteristic adsorption isotherm, indicative of a micro-medium pore structure. The morphological and elemental features of the AC-ZrO_2_/CeO_2_ NCs were measured in FE-TEM. The AC-ZrO_2_/CeO_2_ NCs were polydisperse with obvious shuttle or rod-like structures with <200 nm. The atomic weight ratios of carbon (C, 22.83%), oxygen (O, 42.55%), cerium (Ce, 10.3%), and zirconium (Zr, 24.31%) were verified by the EDS analysis. Nickel/carbon (Ni/C) microspheres were prepared for the removal of methylene blue through photocatalytic degradation [32]. SEM images confirm the spherical shape of Ni/C composites with mean diameter around 3 μm, while the EDS analysis shows the presence of C(93.81% *w*/*w*) and Ni(6.19% *w*/*w*).

### 2.2. Effect of Radiation in the Photocatalytic Materials

**Figure 3:**
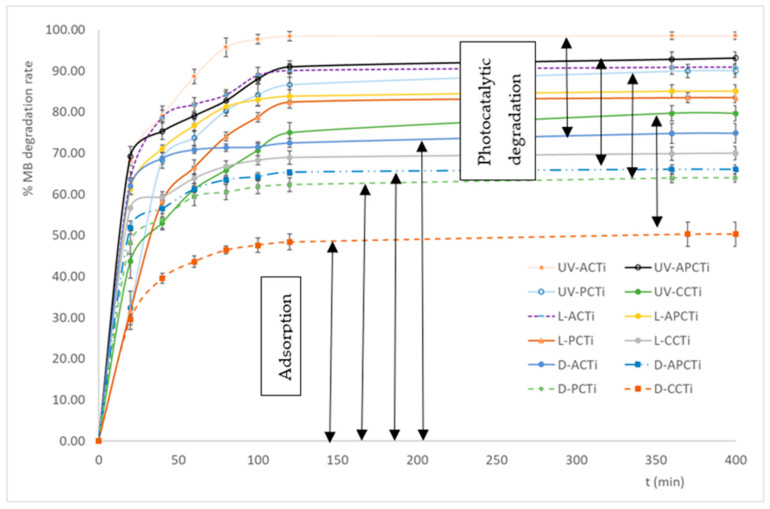
Degradation rate of methylene blue from the photocatalytic materials under UV (UV) radiation, VIS (L) radiation, or dark (D), where AC is the apricot shell carbon, PC the peach shell carbon, and CC the commercial carbon embedded in TiO_2_ (Ti). Each bar represents the mean values ± standard errors.

Using different experimental conditions, i.e., physical light, UV radiation, and dark, on the carbonaceous materials embedded in TiO_2_ solution, the degradation rate of methylene blue (MB) varied (Figure 3). According to the results, the decrease in the degradation MB rate follows the order UV-ACTi > UV-APCTi > L-ACTi ≈ UV-PCTi > L-APCTi ≈ L-PCTi > UV-CCTi > D-ACTi > L-CCTi > D-APCTi > D-PCTi > D-CCTi. It seems that the cationic dye degradation depends on the type of radiation that the experiment takes place preserving all the other parameters constant, i.e., pH, temperature, MB initial concentration, and ratio of photocatalytic material to MB solution. UV radiation enhances the degradation rate of MB from all samples, i.e., ACTi, APCTi, PCTi, and CCTi compared to physical light or darkness, reaching almost 98.5% for UV-ACTi, while darkness reduces the whole phenomenon reaching almost 50.3% for D-CCTi. The removal of MB from aqueous solutions during darkness is due to the adsorption process of MB on the materials, while the removal of MB in an ultraviolet radiation environment is also due to photocatalytic degradation processes. The duration of the phenomenon to reach MB maximum degradation is equal to 100 min. Moreover, comparing the four materials ACTi, APCTi, PCTi, and CCTi, it seems that the order of MB degradation decreases as follows: ACTi > APCTi > PCTi > CCTi, independent of the radiation type, showing that apricot samples degrade MB dye more effectively compared to the other materials. The above analysis follows the specific surface area’s results (S_BET_) and the pore size distribution results, where it seems that the ACTi specific surface area is the highest (342.56 m^2^/g) with total pore volume 19.23 × 10^−2^ cm^3^/g, while the S_BET_ of CCTi is the lowest (26.11 m^2^/g) with total pore volume 58.69 × 10^−3^ cm^3^/g among all samples.

The highest specific surface area with the highest pore volume leads to the highest adsorption of dyes. The photocatalytic degradation of MB dye using a novel ZnO/shrimp shell-derived carbon quantum dots (SS-CQDs) composite under UV light radiation has also been investigated [33]. The ZnO/SS-CQDs composite demonstrated a remarkable degradation efficiency of 94% within 90 min under UV light irradiation using a low catalyst dosage of 20 mg in a 10 ppm MB solution [33]. The large band gap of titanium dioxide (TiO_2_), which is approximately 3.2 eV, means it primarily absorbs ultraviolet (UV) light because this energy is sufficient to excite electrons from the valence band to the conduction band creating an electron–hole pair. This process is principle for photocatalytic activity, where these excited electrons and holes can be used to drive chemical reactions, such as the degradation of pollutants. Photons with energy lower than the band gap, like those in visible light, do not have enough energy to cause this excitation [34].

### 2.3. Effect of pH in the Photocatalytic Materials

**Figure 4:**
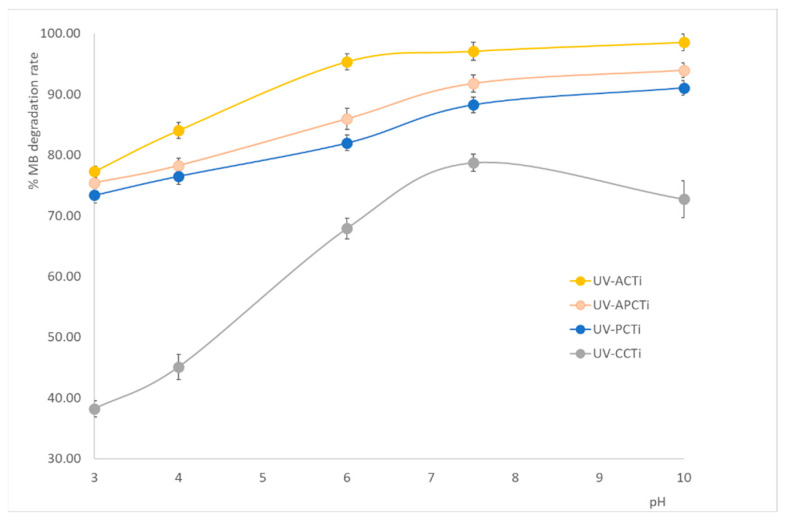
Dependance of pH on the photocatalytic degradation of methylene blue solution by different materials, where each bar represents the mean values ± standard errors.

The dependence of the methylene blue degradation (%) vs. pH is presented in Figure 4. According to the results, all materials presented better methylene blue degradation rates at high pH values. The degradation rate follows the order CCTi < PCTi < APCTi < ACTi. A basic parameter which explains the behavior of MB degradation from the materials is the point of zero charge.

**Figure 5:**
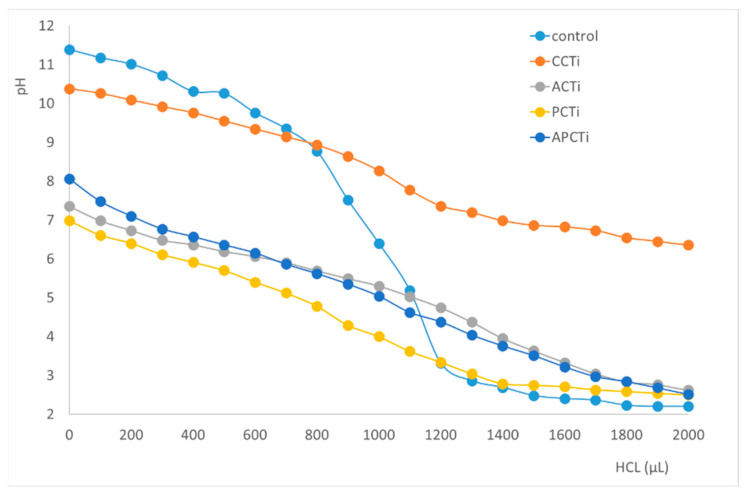
pH of different photocatalytic materials vs. the volume of HCL acid added.

The titration curves of photocatalytic materials and the control treatment to determine the point of zero charge are presented in Figure 5. The intersection of both curves (control and each material separately) are located at pH 8.93 for CCTi, 5.03 for ACTi, 4.38 for APCTi, and 3.34 for PCTi, respectively, indicating the point where the concentration of OH^−^ and H^+^ becomes equal for each material.

Adjusting the pH to be below the catalyst’s point of zero charge (pH_zpc_) often positively charged the surface of the photocatalytic material and increased the degradation by promoting adsorption of negatively charged pollutants and favorable electrostatic interactions. Consequently, a strong electrostatic repulsion is presented between the photocatalytic material and the cationic MB dye. The low levels of MB degradation at this stage were attributed to the π-π interaction, hydrogen bonding, and Van der Waals forces [31]. When the pH of the catalyst is higher than the pH_zpc_, the surface material is negatively charged due the presence of more hydroxide ions in the solution, which deprotonate the surface sites. The negative surface of the catalyst will attract positively charged ions such as methylene blue cationic dye. Alkaline conditions often favor the formation of more hydroxyl radicals, increasing the degradation rate. Generally, the surface of TiO_2_ becomes positively charged in an acidic environment and negatively charged in alkaline conditions due to a pH_zpc_ value equal to 6.2–6.35 [35]. According to Husna et al. [36], the decrease in MB photodegradation activity by mixed oxides TiO_2_/SnO_2_/CeO_2_ under visible light is noticeable in pH solutions between 9 and 11 due to complex formation between OH ions and other ions, which affects the dye–adsorbent interaction. The optimum conditions for the photodegradation of 40 ppm of MB were obtained at pH 7, with 0.2 g of catalyst mass and 120 min of reaction time. Another study [37] has shown that the photocatalytic degradation of methylene blue by flowerlike rutile-phase TiO_2_ film ranged from 11 to 60%, changing the solution pH from 3 to 12 due to the amphoteric behavior of the film.

### 2.4. Effect of Temperature on the Photocatalytic Materials

**Figure 6:**
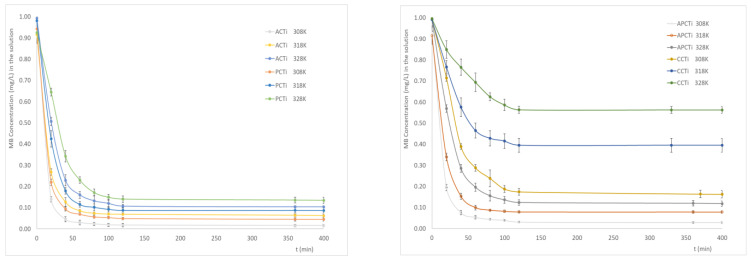
MB concentration in the solution at different time intervals and at different temperatures, i.e., 35, 45, and 55 °C, where each bar represents the mean values ± standard errors.

In the present study, the degradation efficiency of ACTi, APCTi, PCTi, and CCTi was investigated between 35 °C (308 K) and 55 °C (328 K) for a MB solution with initial concentration of 1.0 mg/L and for an irradiation period of 400 min (Figure 6). Comparing the behavior of the four photocatalytic materials, it is observed that MB concentration in the solution decreased due to degradation according to the following order: ACTi-308 < APCTi-308 < PCTi-308 < ACTi-318 < APCTi-318 < PCTi-318 < ACTi-328 < APCTi-328 < PCTi-328 < CCTi-308 < CCTi-318 < CCTi-328.

The results revealed a steady decrease in MB concentration in the solution degraded from 0.02 to 0.10 mg/L for ACTi, 0.03 to 0.12 mg/L for APCTi, 0.04 to 0.14 mg/L for PCTi, and 0.16 to 0.56 mg/L for CCTi, respectively, between 35 and 55 °C.

According to other researchers [38,39,40] at high temperatures, the oxygen saturation level was reduced, hindering the formation of reactive superoxide radicals. This mechanism is needed to capture the photogenerated electrons improving O_2_^−^ formation and subsequently reducing the amount of MB dye in the solution. The degradation of MB in the presence of Zr_2_O thin films under UV radiation were also studied [41]. According to the results, the increase in the temperature from 400 to 550 °C led to a decrease in MB concentration in the solution with the presence of Zr_2_O thin films.

### 2.5. Effect of Initial MB Concentration

**Figure 7:**
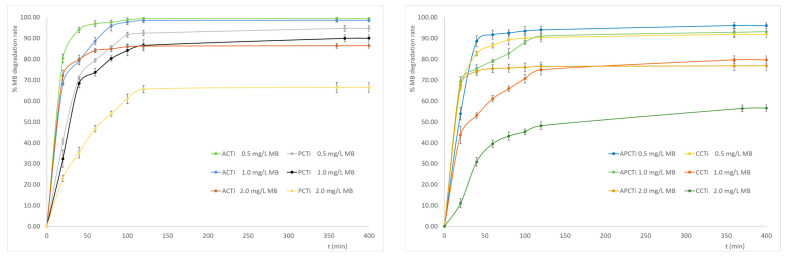
MB degradation rate vs. time at different initial concentration of MB solution, where each bar represents the mean values ± standard errors.

The behavior of the photocatalytic materials towards the differences in the initial concentration of methylene blue is presented in Figure 7. It seems that the MB degradation rate follows the order ACTi-0.5 ≈ ACTi-1.0 > APCTi-0.5 ≈ PCTi-0.5 > APCTi-1.0 ≈ CCTi-0.5 > PCTi-1.0 ≈ ACTi-2.0 > CCTi-1.0 > APCTi-2.0 > PCTi-2.0 > CCTi-2.0. According to the results, it seems that the increase in the initial MB concentration leads to a decrease in MB degradation rate for all the materials, indicating that more time is needed for the organic reactant to be oxidized by the OH^●^ radicals or any other reactive oxidation species produced by the illumination of the photocatalytic materials. The oxidation processes depend on the electron–hole pairs produced by the UV radiation on the carbon-TiO_2_ material and the reaction of these pairs with the oxygen molecules and hydroxide ions in the surface of the carbon-TiO_2_ material. When the quantity of the photocatalytic material remains constant, the amount of oxygen species in the carbon-TiO_2_ surface is the same, so the active sites of the material are the same and can oxidize only a certain amount of MB molecules. The excess amount of MB dye cannot react with the photocatalytic material. Moreover, an excess amount of MB dye may lead to a decrease in the interaction between the photon and the catalyst due to the presence of many dye molecules [42]. Photocatalytic materials derived from apricot shells have shown better photocatalytic behavior compared to materials derived from commercial carbon, peach stone, and mixtures of peach and apricot stones for all the initial MB concentrations. According to the literature [43], the effect of initial BR46 dye concentration on TiO_2_–clay nanocomposite was investigated using different concentrations (20, 30, and 50 mg/L). The results indicated that the highest degradation efficiency was achieved at the lowest concentration (20 mg/L), with the efficiency decreasing as the dye concentration increased. This suggests that lower initial concentrations of the dye enhance the removal efficiency.

### 2.6. Effect of the Initial Quantity of Photocatalytic Material

**Figure 8:**
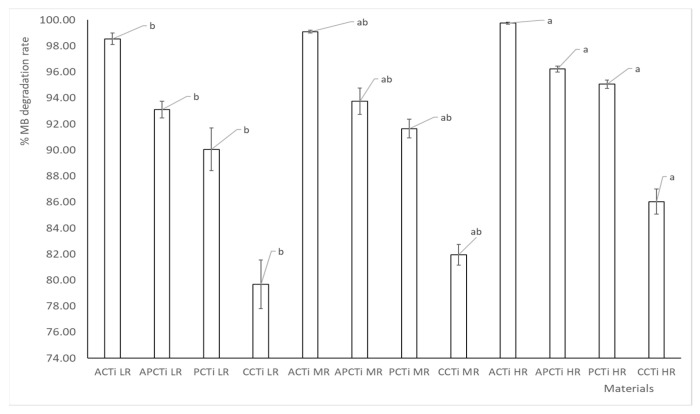
MB degradation rate at different initial amounts of photocatalytic material, with LR: 0.67 g/L MB (lowest ratio), MR: 1.33 g/L MB (medium ratio), HR: 2.00 g/L MB (highest ratio), where each bar represents the mean values ± standard errors. The mean values followed by different superscript letters (a, b) differ significantly (*p* < 0.05).

The effect of the ratio of the photocatalytic material quantity vs. MB concentration (C/MB) was also examined (Figure 8). Comparing photocatalytic materials in the three ratios of C/MB, i.e., 0.67 (LR: lowest ratio), 1.33 (MR: medium ratio), and 2.0 g/L (HR: highest ratio), it is evident that the increase in the ratio of C/MB leads to an increase in the MB degradation rate. The MB degradation rate for every ratio follows the order ACTi > APCTi > PCTi > CCTi. The material derived from apricot shells shows the best MB degradation rate, while commercial photocatalytic carbon presents the worst MB degradation rate, especially at the lowest ratio, i.e., 0.67 g/L C/MB dye concentration. This happens due to the increased active sites of the photocatalytic material, which can produce more electrons due to the UV radiation, helping enable a faster and more effective MB oxidization process, breaking them down into smaller, less harmful molecules and eventually leading to complete mineralization. The increase in the number of active sites correlates with a greater degree of MB degradation as the amount of catalyst is increased. The accumulation of dye on the catalyst surface increases and an increasing amount of photon absorption occurs [42]. The addition of carbon to TiO_2_, producing a new photocatalyst that has enhanced catalytic activity under visible light, was investigated [42]. According to the results, the increase in the photocatalytic amount from 0.05 to 0.15 g per 100 mL of MB solution led to an increase in the MB degradation rate from around 82 to 94%. Another study [36] concerning the photocatalytic degradation of methylene blue by mixed oxides TiO_2_/SnO_2_/CeO_2_ has shown that the increase in the photocatalyst mass enhances the MB degradation until the amount of 0.2 g of photocatalyst per 10 ppm of MB at pH 7.0. However, when the catalyst amount exceeds the optimum condition, the solution becomes turbid, reducing light penetration to the solution and resulting in a decrease in MB degradation. A study concerning the photocatalytic degradation of BR46 dye using a TiO_2_–clay nanocomposite in a rotary photoreactor [43] has shown that the increase in the clay content led to an increase in the BR46 degradation activity up to an optimum value, i.e., 30% *w*/*w* of clay and 70% *w*/*w* of TiO_2_.

### 2.7. Kinetic Analysis of MB Degradation Rate

**Figure 9:**
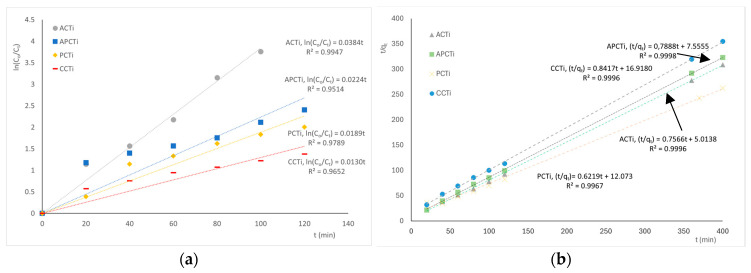
Application of the (**a**) pseudo-first order and (**b**) pseudo-second order model to the experimental data of MB degradation rate on different photocatalytic materials.

**Figure 10:**
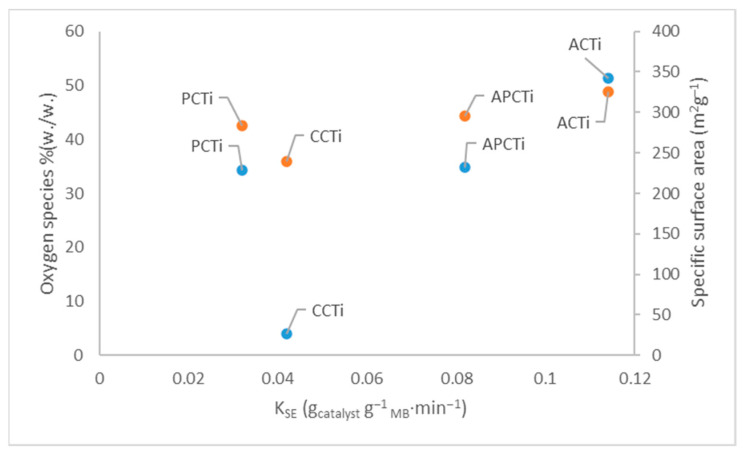
Correlation of oxygen species and specific surface area with the constant, K_SE,_ of the pseudo-second order model for all materials, where the blue values are the specific surface area and the red values the percentage of oxygen species.

The application of different kinetic models to the experimental data of MB degradation rate on photocatalytic materials was presented in Figure 9. According to the results, it seems that the pseudo-second order model best describes the MB degradation rate of the photocatalytic materials due to the best correlation coefficient factors (R^2^ > 0.99). The correlation of the kinetic constant, K_SE_, of the model, as described in Equation (9), vs. the specific surface area values of BET analysis (Table 3) and the percentage of oxygen species from EDS analysis (Table 1) are shown in Figure 10. The removal of MB dye from an aqueous solution depends on material adsorption through the pore size distribution and specific surface area and degradation through the oxidation of MB molecules by the oxygen species. According to the results, it seems that the highest kinetic constant of the second order model, 1.14∙10^−1^ g_catalyst_/g _MB_∙min, corresponds to the highest specific surface area and the highest amount of oxygen species of the ACTi material, indicating the fastest rate at which molecules are adsorbed onto the surface of the material and the strongest interactions (e.g., chemical bonds or ion exchange) between the adsorbate and the adsorbent compared to all the other materials with lower K_SE_ values. Wang et al. [44] reported that MB degradation kinetics of TiO_2_/GO composites follow the pseudo-second order kinetic model with the adsorption capacity to reach 20.25 mg/g_catalyst_ and the constant value of the model to be 3.39 × 10^−2^ g_catalyst_/mg∙min, owing to the strong adsorption capacity of GO and the stacking structure of sheets and nanoparticles. As concluded from the article, the total removal rate of MB is 97.5% after 35 min adsorption and 140 min degradation, which is 3.5 times higher than that of TiO_2_.

The role of specific reactive oxygen species (ROS) in the TiO_2_-photodegradation of MB by employing selective scavenging agents were investigated [45]. The results show that the introduction of a hole scavenger such as EDTA-2Na slightly reduces MB degradation, indicating a low contribution of photogenerated holes (h+), while the hydroxyl radicals, (•OH), and quenching superoxide radicals, (•O_2_^−^), as scavengers significantly lowers MB degradation. The photocatalytic degradation of MB by TiO_2_ predominantly relies on the synergistic interplay between hydroxyl and superoxide radicals and, in a less extent, to photogenerated holes.

### 2.8. Photocatalytic Mechanism of Carbon-TiO_2_ Materials

**Figure 11:**
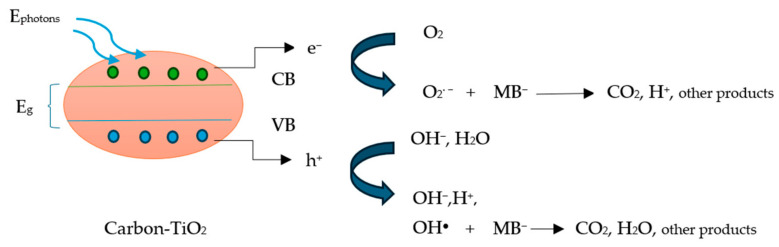
Proposed mechanism of photocatalytic degradation of methylene blue.

The mechanism of photocatalytic degradation of MB from carbon-TiO_2_ materials begins with the UV radiation on the solution (Figure 11). The incident photons with energy higher than that of the catalytic material energy gap led to the production of electrons (e^−^) in the valence band which are transferred to the conduction band, allowing positive holes in the valence band (h^+^) according to Equation (1):

(1) $${TiO}_{2(VB)}+{hv}_{\left(UV\;radiation\right)}\underset{E_{photon}\geq E_{g}}{\to }e_{{TiO}_{2}\left(CB\right)}^{-}+\;h_{{TiO}_{2}(VB)}^{+}$$

where *VB* and *CB* represent the valence band and conduction band, *E_g_* is the energy gap, and *E_photon_* the energy of photons on the catalyst surface.

Then, the conduction band electrons react with the oxygen molecules in the surface of the carbon-TiO_2_ material, producing superoxide anions according to Equation (2):

(2) $$e_{{TiO}_{2}\left(CB\right)}^{-}+\;O_{2}\to O_{2}^{\cdot -}\;\;(superoxide\;anions)$$

The remaining valence band holes react with either water molecules or/and hydroxide ions, leading to different products, i.e., hydroxide anions, hydrogen cations, and hydroxyl radicals, according to Equations (3) and (4):

(3) $$\;h_{{TiO}_{2}(VB)}^{+}+H_{2}O\to {OH}^{-}+H^{+}$$

or/and

(4) $$\;h_{{TiO}_{2}(VB)}^{+}+{OH}^{-}\to {OH}^{●}\;\;\;\;\;\left(hydroxyl\;radicals\right)$$

These superoxide anions and the free hydroxyl radicals react with methylene blue molecules resulting in degrade products, i.e., carbon dioxide, water, and hydrogen molecules, according to Equations (5) and (6):

(5) $${OH}^{●}+{MB}^{-}\to intermediates\;\to \;{CO}_{2}+H_{2}O+{NH}_{4}^{+}+{SO}_{4}^{2-}$$

(6) $$O_{2}^{\cdot -}+{MB}^{-}\to intermediates\;\to \;{CO}_{2}+H_{2}+{NH}_{4}^{+}+{SO}_{4}^{2-}$$

Similar mechanisms are also described elsewhere [33] concerning the photocatalytic degradation of MB from a ZnO/shrimp shell-derived carbon quantum dots (SS-CQDs) composite under UV light radiation. The reactive oxygen species (ROS), including superoxide anions (O_2_^·−^) and hydroxyl radicals (OH^●^), play a crucial role in the degradation of MB dye resulting in the mineralization of MB into by-products such as CO_2_ and H_2_O.

Different carbon-based materials such as fullerene, carbon fiber, carbon nanotube, graphene, and graphene oxide have been used to ameliorate the photocatalytic performance of TiO_2_ [46]. Carbon-based materials present unique optical, electrical, and structural properties [47]. Composites based on carbon/TiO_2_ have shown promising results in tuning the photoactivity of TiO_2_ towards the visible range and minimizing electron charge recombination through the creation of structural defects [48,49]. The extended oxide-carbon interface plays an important role in charge mobility, minimizing fast recombination and ameliorating the photocatalytic activity [48]. Additionally, the carbon matrix prevents the aggregation of TiO_2_ nanoparticles leading to a better dispersion of metal oxides in the carbon material ameliorating the adsorption ability of the composite, maximizing its surface area. Moreover, carbon matrix materials present high adsorption ability, owing to their high surface area and surface functional groups, leading to superior performance in pollutant degradation, hydrogen production, and other photocatalytic processes. Carbon quantum dots (CQDs) are carbon-based nanomaterials derived from natural biopolymers, which relate the electronic properties of carbon-based materials with the optical properties of quantum dots and differentiate them from other carbon nanomaterials or traditional semiconductor quantum dots [46,50]. CQDs are used as carbon-based sensitizers that enhance the photo-response of TiO_2_, suppressing the excitons’ recombination and achieving H_2_ high evolution rates under visible light [46,51]. The role of TiO_2_ in the CQDs/TiO_2_ composite is to function as the semiconducting framework that enhances the photocatalytic role of CQDs [51]. As it seems from Figure 1 and Figure 10, carbon-TiO_2_ material derived from apricot and peach shell exhibited a synergistic effect showing a better photocatalytic efficiency through (i) the availability of high-quality adsorptive-active sites and (ii) a minimization of electron/hole recombination and bandgap tuning. Consequently, a carbon matrix in TiO_2_ composites acts as a multifunctional material, simultaneously functioning as an adsorbent for pollutants, a support preventing TiO_2_ particle aggregation, an electron mediator to separate charges, and even a photosensitizer (in CQDs), all working synergistically to enhance visible light absorption, suppress electron–hole recombination, and boost overall photocatalytic efficiency for environmental or energy applications.

## 3. Materials and Methods

### 3.1. Production of the Materials

Apricot (*Prunus armeniaca* L.) and peach (*Prunus persica*) kernels were transferred to the laboratory from a Greek juice industry in Naoussa, Greece. Kernels were then properly washed to remove any residues. They were then stored in room temperature for 24 h and then placed in the oven at 103 ± 2 °C for 2 h to dry. Finally, they were placed in a urine collection container at 4 °C for further use.

The received apricot and peach kernels are crushed with a crusher, then they are ground in a blender, and finally sieved with a sieve with a pore diameter of 1 mm. Two samples were prepared from the obtained powders derived from apricot and peach kernels. The samples were placed in ceramic dishes and introduced into a horizontal high-temperature furnace in the presence of inert nitrogen gas with a flow of 500 mL/min. The mass of each sample was equal to 7.0 g.

The temperature program of the oven for the carbonization of the materials was initially 20–220 °C with a temperature increase of 10 °C/min, staying at the temperature of 220 °C for 15 min, increasing to 440 °C at a rate of 10 °C/min, and staying at 440 °C for 15 min. Then, it increased to 660 °C at the same rate and remained constant at 660 °C for 15 min. Finally, it increased to 850 °C at a rate of 10 °C/min and remained constant at 850 °C for 30 min. The weight of the samples was measured before and after carbonization. The temperature program of the carbonization process is based on the technical characteristics of the oven and the type of the final product. More specifically the gradual increase in temperature is a necessity for an oven with temperature range up to 1100 °C so as not to cause severe damage to the oven. Furthermore, carbonization is a process where the materials will be degraded at temperatures between 300 and 1000 °C under an inert atmosphere of nitrogen or argon gas flow [52,53]. The degradation of lignocellulosic materials such as apricot and peach shells follows the next steps: hemicellulose degradation between 200 and 350 °C, cellulose degradation between 200 and 500 °C, and finally lignin degradation between 150 °C and 900 °C [54]. Low degradation rates (approximately 10 °C/min) will help the gradual structural changes in the lignocellulosic materials more effectively, while the final temperature of up to 900 °C and time will lead to porous materials with a high degree of carbon content. Generally, higher temperatures increase carbon content, aromatization, and surface area but decrease yield, while longer times further ameliorate these properties.

The photocatalytic materials are then prepared, i.e., 2.5 g of each pyrolyzed material is mixed with a TiO_2_ solution (2.5% *w*/*v*). Titanium dioxide pigment powder was bought for the above specific purpose (Nurture Soap, Huntington, IN, USA, 99.9% purity). For comparison purposes, commercial activated charcoal powder (Labchem, Oxford, UK, CAS7440-44-0 EC) and pyrolyzed carbon derived from the mixture of apricot and peach kernels in a proportion of 50/50% *w*/*w* were also used and subjected to the same process. The respective solution was stirred for 2 h in a magnetic stirrer at 300 rpm and then ultrasound was used for 30 min. The solutions were then transferred to the air-dryer at 120 °C for 8–9 h and then they were placed in an oven at 400 °C for 2 h in the presence of air [55].

### 3.2. Characterization of the Adsorbed and Photocatalytic Materials

SEM-EDS studies of the produced materials were performed with a JEOL JSM-IT500 scanning electron microscope (SEM), JEOL Ltd., Boston, MA, USA and simultaneously elemental analysis was performed with energy dispersive X-ray spectrometry (EDS) using an Oxford Instruments x-Act detector from Oxford Instruments, High Wicombe, UK. Before these analyses, the samples with TiO_2_ were coated with a thin layer of Au by sputtering technique using AGAR Sputter Coater apparatus (AGAR Scientific Company, Rotherham, UK).

The specific surface area and porosity of the produced materials were analyzed using the Tristar II plus surface area and porosity analyzer. The carrier gases were helium and nitrogen. The samples were weighed, placed in a sample container in the analyzer, and degassed separately for 24 h at 400 °C. The weight of the samples ranged from 0.14 to 0.2 g. They were then allowed to cool down before being stored under nitrogen gas at a pressure of 5 mmHg for 24 h. Characterization in terms of specific surface area, pore volume, and pore diameter of the obtained materials was determined by N_2_ adsorption–desorption at −196 °C using the BET method. The micropore volume was calculated by using t-plot micropore volume. The pore size distribution was determined by using the Barrett–Joyner–Halenda (BJH) model [56].

### 3.3. Influence of Radiation in the Produced Materials

The experiments are performed either in the presence of UV radiation, VIS radiation, or in the dark. First, 0.01 g of each photocatalytic material is introduced into 25 mL conical flasks with 15 mL of methylene blue dye solution at a concentration of 0.001 g/L. Then, they were shaken on an incubator shaker at 180 rpm at 30 °C under different conditions of light, darkness, and UV radiation, respectively, for different time intervals. At regular intervals, they are centrifuged in 20 mL plastic falcon tubes at 9000 rpm for 10 min. The absorbance value of MB photodegraded or adsorbed by the activated carbon-TiO_2_ composites was measured using a UV-Vis spectrophotometer (UV-1900i Shimadzu, Santa Clara, Ca, USA) at 664 nm. From these values, the photodegradation rate of methylene blue can be calculated. The results are expressed as photodegradation percentage of MB solution by the photocatalytic materials at regular time intervals, expressed by the following mathematical type:

(7) $$\%MB\;degradation\;rate=\frac{C_{o}-C_{t}}{C_{o}}\;\times 100$$

where *C_o_* is the initial concentration of the dye in the solution and *C_t_* the concentration of the dye after radiation at different time intervals, *t* (min). The optimal radiation conditions used to study the effect of the remaining parameters are found.

### 3.4. Influence of pH Solution

The experiments were carried out at pH 3, 4, 6, 7.5, and 10. The solutions contain an amount of 0.01 g of each photocatalytic material in 15 mL of a solution of the pigment concentration of 0.001 g/L MB. The pH adjustment of the solutions was carried out by adding 0.1 M sulfuric acid (H_2_SO_4_) for an acidic environment and 0.1 M sodium hydroxide (NaOH) for a basic environment. The pH was measured using a pH meter. The solutions were stirred for 24 h in an incubator shaker at 180 rpm at 30 °C with UV radiation, then placed in plastic falcon tubes with screw caps and centrifuged for 10 min at 9000 rpm. They are then placed in the UV-1900i spectrophotometer to measure the MB absorption at specific wavelengths. The results are expressed as MB degradation percentage by the materials in 24 h.

The point of zero charge of each material was determined according to the acid-base titration method by Bocris et al. [57]. Specifically, 25 mL of 0.004 M KOH and 0.01 M KCL solution was added to 100 mL of Erlenmeyer flask and mixed with 0.3 g of each photocatalytic material. The solution was left in agitation for 12 h. Then, the mixture was titrated by adding 100 μL aliquots of a 0.1 M HCL solution. The changes in pH as a function of the volume of HCL solution added to the flask was recorded. Separately, the same procedure was carried out without the photocatalytic material acting as a control treatment. The point of zero charge of the photocatalytic material located at the intersection of the curve of the control with the neutralization curve of each material solution was determined graphically.

### 3.5. Influence of Temperature

The experiments were carried out at three different temperatures of 35, 45, and 55 °C. The solutions contain 0.01 g of pyrolyzed carbon with titanium oxide in 15 mL of 0.001 g/L MB solution. The samples are placed under agitation in an incubator shaker at 180 rpm with UV radiation for different time intervals. They are then placed in plastic falcon-type tubes, where centrifugation is performed at 9000 rpm for 10 min. They are then placed in the spectrophotometer where the absorbance is measured. The results are expressed as MB concentration in the solution at regular time intervals.

### 3.6. Influence of the Initial MB Concentration

The experiments were performed at different MB concentrations of 0.0005, 0.001, and 0.002 g/L. The solutions contain an amount of 0.01 g of pyrolyzed carbon–titanium oxide in 15 mL of MB dye solution. The samples are subjected to shaking in an incubator shaker at 180 rpm and 30 °C with UV radiation for different time intervals. They are then placed in plastic falcon tubes and centrifuged at 9000 rpm for 10 min. They are then placed in the spectrophotometer where the absorbance is measured.

### 3.7. Influence of the Initial Quantity of Photocatalytic or Adsorbent Material

The experiments were performed on different amounts of pyrolyzed carbon-TiO_2_: 0.67, 1.33, and 2.0 g/L MB. The solutions contain different amounts of pyrolyzed carbon with titanium oxide and were mixed with 15 mL of MB solution 0.001 g/L, stirred in an incubator shaking table at 180 rpm, 30 °C with UV radiation, and finally were measured by UV/VIS spectrophotometer at different time intervals.

### 3.8. Kinetic Analysis of the Materials

The kinetic rate constant for the degradation of ΜΒ in the produced materials was calculated using a pseudo-first order and a pseudo-second order kinetic model [44] expressed by the most recent equations:

Pseudo-first order equation: ln(C_o_/C_t_) = k_FE_t (8)

(9) $$\mathrm{Pseudo-second}\;\mathrm{order}\;\mathrm{equation}:\;\frac{t}{q_{t}}=\frac{1}{K_{SE}q_{e}^{2}}+\frac{t}{q_{e}}$$

where C_o_ and C_t_ are the initial MB ion concentration and MB ion concentration at time t, respectively, in g_MB_ L^−1^; q_e_ and q_t_ its value at equilibrium and at time t (g_MB_·g_catalyst_^−1^); and k_FE_ and K_SE_ the pseudo-first order (min^−1^) and the pseudo-second order (g_catalyst_·g_MB_^−1^·min^−1^) rate constants.

### 3.9. Statistical Analysis

The experimental data and standard errors were analyzed using Microsoft Excel (Microsoft 365), while the statistical analysis (ANOVA with a post hoc Tuckey’s honest significant difference test) was performed with the help of JASP 0.19.1.0 [57]. When the *p*-value corresponding to the F-statistic of a one-way ANOVA is less than 0.05, it suggests that one or more treatments are significantly different (indicated by different lowercase letters in the figures); otherwise, the treatments show insignificant differences (indicated by the same letters in the figures).

## 4. Conclusions

Efficient photocatalysts, based on agro-food by-products and TiO_2_, were synthesized using apricot and peach shells. The resulting carbon-doped TiO_2_ derived from apricot shell (ACTi) exhibits the highest specific surface area (342.56 m^2^/g), the lowest mean pore diameter (22.45 Å), and the highest percentage of oxygen species (48.8% *w*/*w*) according to EDS analysis compared to all the other materials. The photocatalytic efficiency was significantly enhanced, achieving approximately 98.5% degradation of methylene blue under UV light when apricot shell carbon-doped TiO_2_ was used as the photocatalyst instead of commercial charcoal-doped TiO_2_. Different parameters were examined showing that all materials presented better methylene blue degradation rates at alkaline pH values and 35 °C. Photocatalytic materials derived from apricot shell have shown better photocatalytic behavior at different initial MB concentrations compared to all the other materials. The increase in the ratio of photocatalytic material/MB solution leads to an increase in the MB degradation rate due to the increase in the active sites of catalyst. The MB degradation rate follows the order ACTi > APCTi > PCTi > CCTi. The kinetic model that best describes the experimental data of MB degradation from the photocatalytic materials is the pseudo-second order model. Future work will involve scaling up the synthesis of the catalyst and evaluating its performance using bed reactors for industrial processes.

## Figures and Tables

**Table 1:** EDS analysis (*w*/*w*) of the produced materials with TiO_2_ addition.

Samples	C	O	Κ	Si	Ca	Na	Al	Fe	Ti	Cl	Mo	C/O
CCTi ^a^	3.8	35.9	3.8	1.7	0.8	–	1.6	1.5	50.7	0.3	–	0.11
CCTi ^b^	74.0	11.5	4.4	1.5	–	1.0	0.9	0.8	3.2	1.8	0.9	6.43
ACTi ^a^	3.9	48.8	–	–	–	–	–	–	47.3	–	–	0.08
ACTi ^b^	81.7	16.6	0.5	–	–	–	–	–	1.2	–	–	4.92
PCTi ^a^	11.5	42.5	–	–	–	–	–	–	46.0	–	–	0.27
PCTi ^b^	84.5	14.5	0.6	–	0.2	–	–	–	0.2	–	–	5.83
APCTi ^a^	2.6	44.3	0.2	–	–	–	–	–	52.9	–	–	0.06
APCTi ^b^	83.0	15.7	1.3	–	–	–	–	–	1.7	–	–	5.29

^a^ refers to the small particles of TiO_2_ which fulfill carbon structure (white color in the SEM images, Figure 1) and ^b^ refers to the pyrolyzed carbon structure of each material (gray cavities in the SEM images, Figure 1).

**Table 2:** EDS analysis (*w*/*w*) of the impurities in the produced materials with TiO_2_ addition.

Samples	C	O	Fe	Ca	K	Mg	Si	Al	Na	P	S	Ti	Cl
CCTi	29.5	36.5	0.6	0.5	2.8	0.2	1.5	1.5	0.5	–	0.2	26.1	0.2
ACTi	33.4	39.8	–	2.7	0.4	–	1.1	–	–	2.0	–	20.6	–
PCTi	36.1	32.3	–	–	0.7	–	–	–	–	–	–	31.0	–
APCTi	5.8	47.2	–	–	0.4	–	–	–	–	–	–	46.6	–

**Table 3:** Characteristics of pore structure, i.e., SBET: specific surface area; Vtot: total pore volume; Vmicro: micropore volume; daver.p.d.: adsorption average pore diameter; and d_BJH_, _aver.p.d._: BJH adsorption average pore diameter.

Samples	S_BET_ (m^2^/g)	V_tot_ (cm^3^/g)	V_micro_ (cm^3^/g)	d_aver.p.d._ (Å)	d_BJH aver.p.d._ (Å)
CCTi	26.11	58.69 × 10^−3^	52.88 × 10^−4^	89.89	124.83
ACTi	342.56	19.23 × 10^−2^	98.14 × 10^−3^	22.45	54.28
PCTi	228.88	13.34 × 10^−2^	68.44 × 10^−3^	23.32	68.74
APCTi	231.99	13.83 × 10^−2^	65.43 × 10^−3^	23.84	64.30

## Data Availability

The original contributions presented in this study are included in the article/Supplementary Materials. Further inquiries can be directed to the corresponding author.

## Supplementary Materials

The following supporting information can be downloaded at: https://www.mdpi.com/article/10.3390/molecules31020300/s1, Figure S1: SEM images of the adsorbent materials: (a,b) pyrolyzed carbon derived from apricot shell (AC), (c,d) pyrolyzed carbon derived from peach shell (PC), and (e,f) pyrolyzed carbon derived from apricot and peach shell in a proportion of 50/50 *w*/*w* (APC) at two different magnifications 50× and 10×; Figure S2: (a) The N_2_ adsorption–desorption isotherm and (b) the pore size distribution (PSD) based on the BJH method of the prepared samples; Table S1: EDS analysis (*w*/*w*) of the produced materials with and without TiO_2_ addition; Table S2: Characteristics of pore structure, i.e., SBET: specific surface area, Vtot: total pore volume, Vmicro: micropore volume, daver.p.d.: adsorption average pore diameter, and dBJH, aver.p.d.: BJH adsorption average pore diameter.

## Abbreviations

The following abbreviations are used in this manuscript:

BET	Brunauer–Emmett–Teller
SEM	Scanning electron microscopy
EDS	Energy dispersive spectroscopy
MB	Methylene blue
ACTi	Apricot carbon–titanium oxide
APCTi	Apricot-peach carbon–titanium oxide
PCTi	Peach carbon–titanium oxide
CCTi	Commercial charcoal-Titanium oxide
AC	Apricot carbon
APC	Apricot-peach carbon
PC	Peach carbon
UV	Ultraviolet
ANOVA	Analysis of variance
BJH	Barrett–Joyner–Halenda
ROS	Reactive oxygen species
EDTA-2Na	Ethylenediaminetetraacetic acid disodium salt
TPP	Total pore volume
TMV	t-plot microporous volume
