# Supplementary material for: Production and Characterization of Novel Photocatalytic Materials Derived from the Sustainable Management of Agro-Food By-Products
Source: Molecules. 2026 Jan 14;31(2):300. doi: 10.3390/molecules31020300 (PMC12844144; doi:10.3390/molecules31020300)
Supplement: Supplementary file 1 [file molecules-31-00300-s001.zip › molecules-4062563-supplementary.pdf]

Production and characterization of novel photocatalytic materials derived from the sustainable management of agro-food by-products

Christina Megetho Gkaliouri, Eleftheria Tsampika Laoudikou, Zacharias Ioannou*, Sofia Papadopoulou, Vasiliki Anastasia Giota, Dimitris Sarris

Laboratory of Physico-Chemical and Biotechnological Valorization of Food By-Products, Department of Food Science and Nutrition, School of the Environment, University of the Aegean, Mitrop. Ioakeim 2, Myrina, 81400 Lemnos, Greece; fnsd21008@fns.aegean.gr (C.G.); fns20075@fns.aegean.gr (E.-T.L.); sofiapdl244@outlook.com (S.P.); siliagiota@gmail.com (V.-A.G.); dsarris@aegean.gr (D.S), *Correspondence: zioan@aegean.gr (Z.I.)

Supplementary material

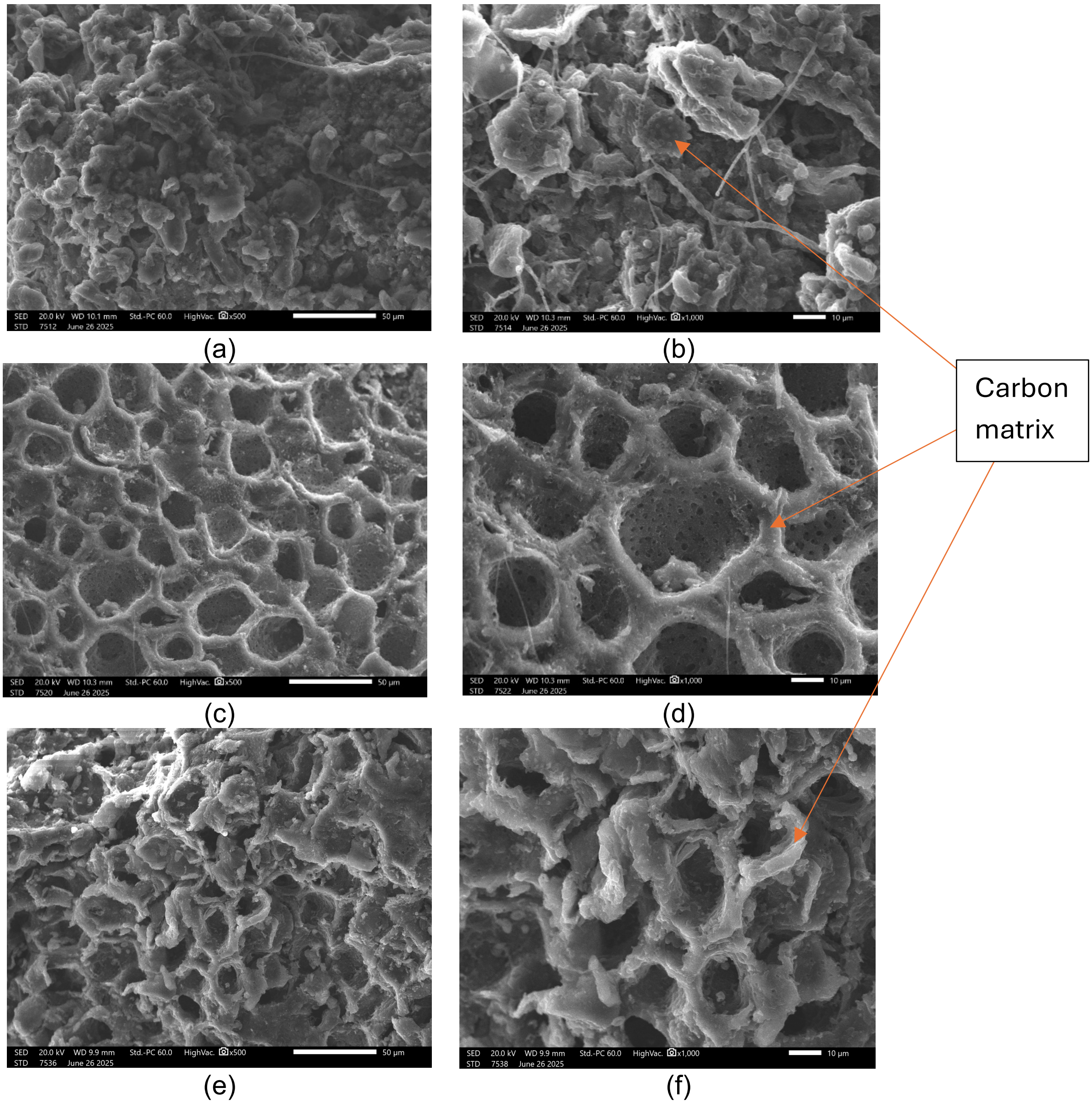

Figure S1. SEM images of the adsorbent materials: a,b) pyrolyzed carbon derived from apricot shell (AC), c,d) pyrolyzed carbon derived from peach shell (PC), e,f) pyrolyzed carbon derived from apricot and peach shell in a proportion of 50/50 w/w (APC) at two different magnifications 500× and 1000×.

Table S1. EDS analysis (w/w) of the produced materials without TiO_2 addition

Samples	C	O	K	Ca	S	P	Mg	C/O
AC	88.0	7.3	2.5	0.4	0.7	0.7	0.3	12.1
PC	91.4	6.9	1.4	--	0.2	0.1	--	13.2
APC	89.3	6.3	4.1	--	--	0.3	--	14.2

Table S2. Characteristics of pore structure, i.e., S_{BET}: specific surface area, V_{tot}:total pore volume, V_{micro}: micropore volume, $d_{aver.p.d.}$: adsorption average pore diameter, $d_{BJH,\ aver.p.d.}$: BJH adsorption average pore diameter

Samples	S_{BET} (m²/g)	V_{tot} (cm³/g)	V_{micro} (cm³/g)	$d_{aver.p.d.}$(Å)	$d_{BJH\ aver.p.d.}$(Å)
AC	6.04	$11.35x10^{-3}$	$10.86x10^{-4}$	75.16	159.13
PC	3.71	$8.39x10^{-3}$	$10.03x10^{-4}$	90.53	216.47
APC	2.17	$8.31x10^{-3}$	$4.85x10^{-4}$	153.50	194.93

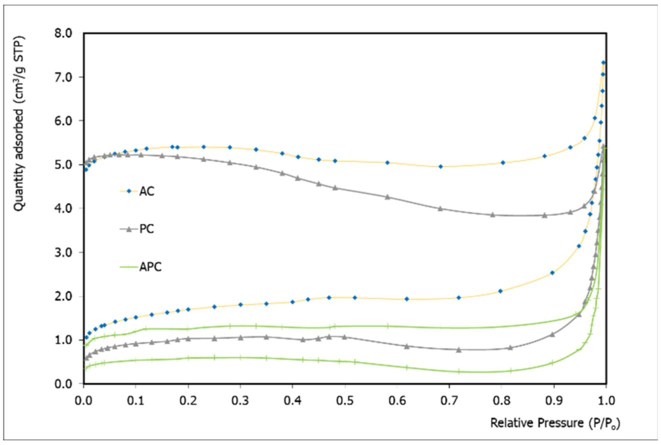

(a)

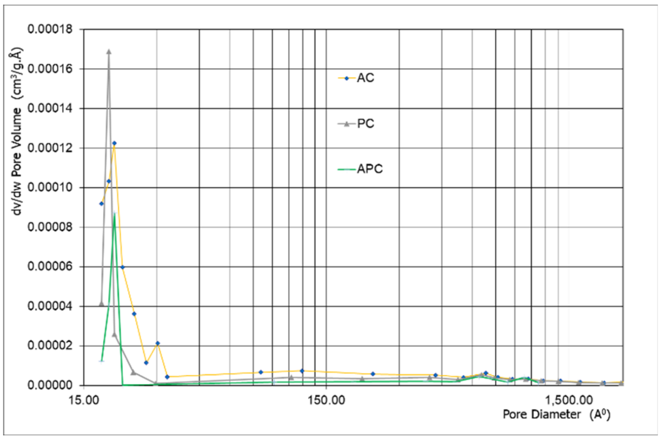

(b)

Figure S2. (a) The N_2 adsorption-desorption isotherm and (b) the pore size distribution (PSD) based on the BJH method of the prepared samples
